# The effects of total hip arthroplasty in treating hip bony fusion in young and middle-aged patients with ankylosing spondylitis

**DOI:** 10.1186/s13018-019-1288-5

**Published:** 2019-08-08

**Authors:** Hong-Zhang Guo, Chen-Xu Yang, Zhao-Peng Tang, Cheng-Xiang Wang

**Affiliations:** Department of Orthopaedics, Gansu Provincial Hospital of TCM, Lanzhou, 730050 China

**Keywords:** Ankylosing spondylitis, Artificial total hip arthroplasty, Hip joint function, Curative effects

## Abstract

**Background:**

This study aims to investigate the curative effects of total hip arthroplasty (THA) in treating hip bony fusion for young and middle-aged patients with ankylosing spondylitis (AS).

**Methods:**

The clinical data of 26 young and middle-aged patients with AS (31 coxae), who were treated with THA and followed-up for more than 3 years in the period between February 1998 and May 2013, were retrospectively analyzed. Among these patients, 22 patients were male (25 coxae) and 4 patients were female (6 coxae). Patients’ age ranged within 19–50 years old, with an average of 31.5 years old. The intervals from arthroplasty to the occurrence of hip joint lesions caused by AS ranged within 2–26 years, with an average of 11.2 years. The average Harris score before the surgery was 19.0 ± 11.5 points.

**Results:**

Femoral proximal cleavage fracture occurred in one coxa during the surgery and was fixed by the steel wire cerclage. Sciatic nerve traction injury occurred in one coxa after the surgery, which recovered after 6 months. Posterior hip dislocation occurred in one coxa and was immediately treated with manual reduction. All patients were followed-up, and follow-up duration ranged within 36–123 months, with an average of 46.5 months. In the last follow-up, the average Harris score was 87.1 ± 13.1 points, total passive range of motion was 215.0 ± 22.0°, and passive range of flexion was 90.8 ± 9.3°. All these indexes significantly increased compared with pretreatment (*P* < 0.01). A periacetabular radiolucent line occurred in one coxa with a width of < 2 mm, and no femoral radiolucent line was found during follow-ups in any patient. Heterotopic ossification occurred in four coxae.

**Conclusion:**

THA treatment for hip bony fusion caused by AS can achieve satisfactory hip function recovery and excellent prosthesis survival rate.

## Introduction

Ankylosing spondylitis (AS) is a kind of unexplained chronic inflammatory disease that predominantly affects the axial joints. Overall, the prevalence of AS is estimated to be between 0.1 and 1.4% worldwide, with nearly 90% patients having the HLA-B27 allele [1]. In the early stage, AS treatment includes disease modifying anti-rheumatic drugs (DMARDs) such as sulfasalazine and methotrexate. Peripheral joints are involved in approximately 70% patients with AS, and the most common joints involved are the hip joints (in 25–50% of patients) [2]. In the late stage, some patients may develop hip bony fusion and even hip joint deformity and dysfunction, which seriously affect a patient’s quality of life. Standard treatment option for advanced hip disease is total hip arthroplasty (THA). Indications of THA are refractory pain, disability, and radiologic evidence of damage in hips, regardless of age [3].

From February 1998 to May 2013, a total of 26 young and middle-aged patients with AS combined with hip joint lesions (31 coxae) underwent THA in our hospital and were followed-up for more than 3 years. All of these patients achieved good medium-term curative effect. Details are reported as follows.

## Clinical data

### General information

This study enrolled 22 male (25 coxae) patients and 4 female (6 coxae) patients. The ages of these patients ranged within 19–50 years old, with an average of 31.5 years old. The intervals from arthroplasty to the occurrence of hip joint lesions caused by AS ranged within 2–26 years, with an average of 11.2 years. Flexion stiffness occurred in 24 coxae (30–85°, average of 60.5°). Flexion abduction stiffness occurred in four coxae (45–95°, average of 72.3°). Flexion adduction-shortening stiffness occurred in three coxae (20–35°, average of 22.5°). Furthermore, 5 patients completely lost their life self-care ability, 16 patients could walk with the assistance of a walker or crutch, and the remaining patients could limp. The average Harris score before the surgery was 19.0 ± 11.5 points. X-ray examination revealed that bony fusion between the acetabulum and femoral head occurred, and the anatomical relationship between the femoral head and cotyle was completely destroyed. In nine patients (10 coxae), the bone cortex became thinner and osteoporosis occurred.

### Surgical methods

**Fig. 1:**
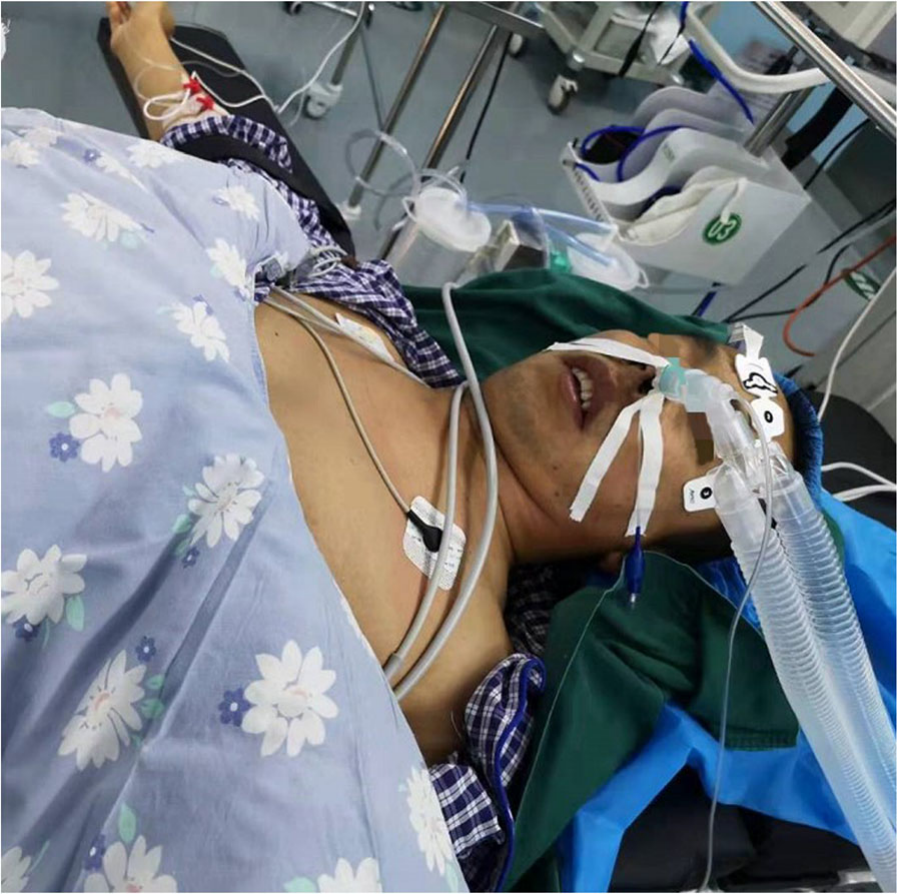
The patient under-went nasal endotracheal intubation because of difficulty in tracheal intubation

Five patients underwent bilateral THA. Among them, three patients underwent one-stage bilateral THA and two patients adopted the two-stage procedure. Furthermore, 23 patients underwent general anesthesia with tracheal intubation, because their lumbar vertebrae were affected by AS, and intraspinal anesthesia could not be performed. Among them, eight patients underwent nasal endotracheal intubation because of difficulty in tracheal intubation (as shown in Fig. 1). The remaining patients all underwent intraspinal anesthesia. Six coxae were treated via the Smith-Peterson approach, 2 coxae were treated via the Hardinge approach, and 23 coxae were treated via the Moore approach. For patients with hip flexion deformity, soft tissues in contracture status surrounding the joints were initially relaxed. This includes the iliopsoas muscle, the musculus rectus femoris, and the iliotibial band. For patients with severe hip flexion adduction, the femoral adductor could be cut off based on the above relaxation. After exposure, the femoral neck was cut off at the site 1.0–1.5 cm above the trochanter minor. When the boundary between the acetabulum and femoral head was determined, the original femoral osseous substance was removed using a bone knife. Then, a mortar was made at the original site of the true acetabulum using an acetabular reamer. The prostheses of the acetabular cup and femoral stem were made of non-bony cement material and were fixed using the press-fit technique. Furthermore, 22 acetabular cups were treated with 2–3 pieces of screw to strengthen the fixation of the acetabulum prosthesis. Ceramic-ceramic interface was used in 3 coxae, ceramic-polyethylene interface was used in 2 coxae, and the CoCrMo alloy-polyethylene interface was used in 26 coxae. The diameter of the femoral head prosthesis was 28 mm in 26 coxae, 32 mm in 3 coxae, and 36 mm in 2 coxae.

### Postoperative measures

After surgery, routine measures were performed to prevent infection and lower extremity deep vein thrombosis. The drainage tube was removed after 48 h. The affected limb was placed in a neutral position of hip abduction (30°), knees were bent at 30°, and a T-shaped pillow was placed between the two lower limbs when patients were in a lying position for 2 weeks. Two days after the surgery, patients were orally given 25 mg of the non-steroidal anti-inflammatory drug indomethacin, twice daily, for 4 weeks, in order to prevent heterotopic ossification. On the day of the surgery, patients were encouraged to undergo femoral quadriceps exercise and ankle movement; and on postoperative day 1, patients were instructed to start walking with the aid of crutches. In the second month, patients walked with part weight-bearing with the aid of a single crutch and walked without a crutch in the third month.

### Statistical methods

The statistical analysis of data was conducted using statistical software SPSS 16.0. All data were expressed as mean ± standard deviation (x ± SD). Intergroup comparison was conducted using paired *t* test. Inspection level was *α* = 0.05.

## Results

**Fig. 2:**
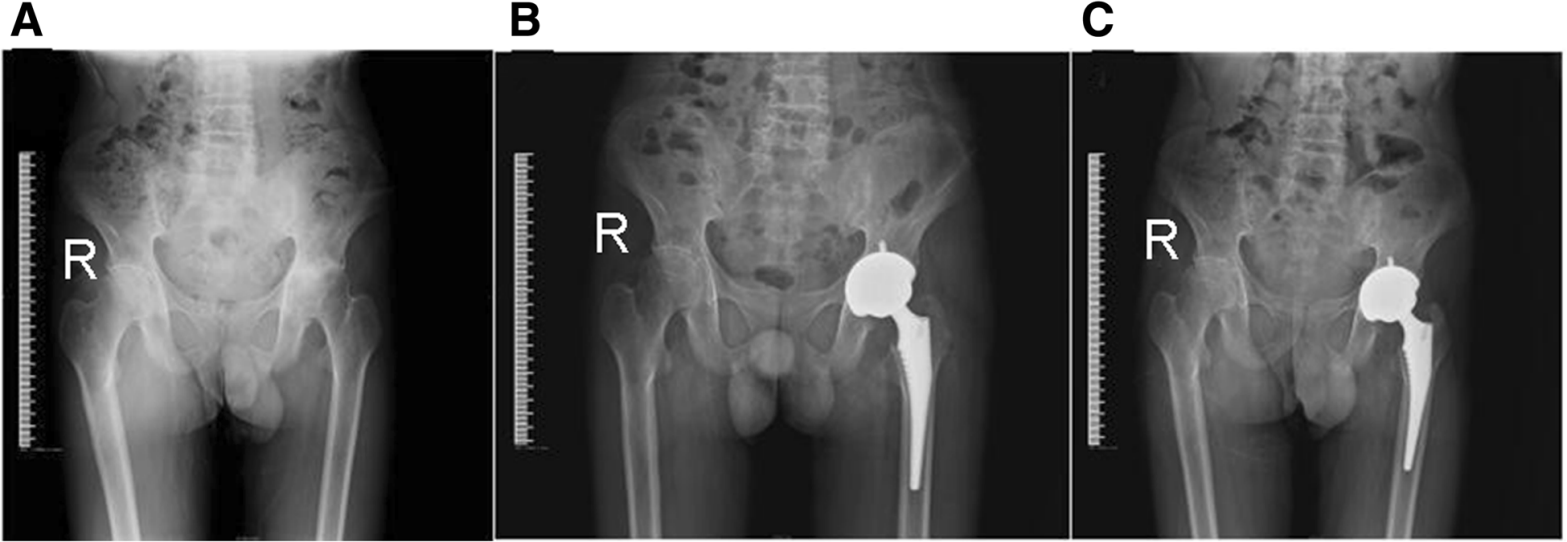
X-ray films of 2 typical cases before and after operation. **a** Male, 39 years old, preoperative pelvic X-ray film. **b** Male, 39 years old, postoperative 2 days, the pelvic normal X-ray. **c** Male, 39 years old, postoperative 5 year pelvic X-ray film

**Fig. 3:**
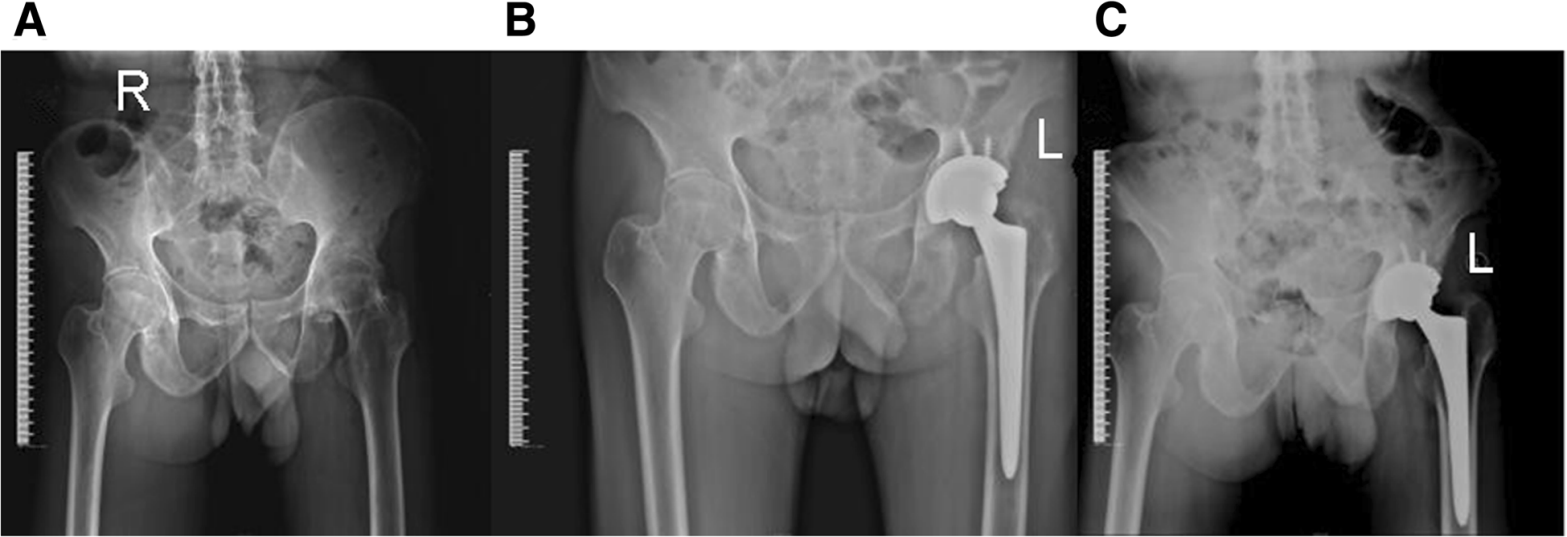
X-ray films of 1 typical cases before and after operation. **a** Male, 20 years old, preoperative pelvic X-ray film. **b** Male, 20 years old, postoperative 5 days, the pelvic X-ray film. **c** Male, 20 years old, postoperative 4 year pelvic X-ray film

All incisions healed by first intention. Complications such as lower extremity deep vein thrombosis, pulmonary embolism, and deep infection did not occur. All patients were followed-up, and follow-up duration ranged within 36–123 months, with an average of 46.5 months. Femoral proximal cleavage fracture occurred in one coxa during the surgery and was fixed with steel wire cerclage, which healed after 3 months. Sciatic nerve traction injury occurred in one coxa after the surgery. After half a year of rehabilitation exercise, the damaged neural function fully recovered. Posterior hip dislocation occurred in one coxa and was immediately treated with manual reduction. During the last follow-up, the average Harris score was 87.1 ± 13.1 points, total passive range of motion was 215.0 ± 22.0°, and passive range of flexion was 90.8 ± 9.3°. All these indexes significantly increased compared with pretreatment data (*P* < 0.01). Hip joint functions in patients significantly improved compared with pretreatment level. For patients with abnormal gait before the surgery, due to the poor strength of the mesoglutaeus, two patients slightly swung when they walk. In the remaining patients, the gaits returned to normal. X-ray examination revealed that all prostheses presented bony in-growth and were stable. A periacetabular radiolucent line occurred in one coxa, with a width of < 2 mm. No radiolucent line was found in other prostheses of the acetabulum and femur. Furthermore, no femoral radiolucent line was found during follow-ups in any patients. Heterotopic ossification occurred in three patients (four coxae), and all were Brooker grade II. The incidence was 12.9% (4/31). No special treatment was given to these patients. After the surgery, hip pain occurred in five patients (six coxae) when the patients walked. Anti-AS drugs and non-steroidal anti-inflammatory drugs were required. The remaining patients did not experience any pain in their operated coxae (as shown in Figs. 2 and 3).

## Discussion

AS is a chronic inflammatory disease that mainly affects the axial skeleton and joints. It often starts from invasion of the sacroiliac joint and is often combined with hip joint dysfunction. In AS patients with hip joint involvement, the range of motion of the hip joint decreases in different degrees and 40% of patients have hip joint stiffness [4].

### Timing of surgery and perioperative management

Joshi et al. considered that in order to reduce the number of repeated operations, THA for AS patients was performed after stabilizing the condition [5]. Hence, patients have to endure great pain waiting for the best time for THA. Under this condition, patients suffer from the involvement of multiple joints and most of their hip joints develop bony fusion, severe flexion deformity, and osteoporosis. Furthermore, their quality of life is severely affected for a long period of time, and the difficulty degree of the operation is increased. Most of patients in the early stage receive THA many years after hip joint stiffness. Therefore, the postoperative rehabilitation period is long, and functional recovery is not ideal. Due to the development of artificial joint materials and the improvement of surgical ideas and techniques, patients in the late stage undergo THA as soon as hip joint bony fusion emerges and if their physical conditions can tolerate the surgery. Therefore, the joint range of motion and self-evaluation are better than in patients during the early stage.

Infection is a catastrophic complication of THA surgery, comprehensive evaluation should be carried out before the operation, and infectious diseases should be excluded. A study conducted by Reveille [6] revealed that in AS patients in the inflammatory stage, inflammatory markers such as C-reactive protein (CRP), IL-6, and erythrocyte sedimentation rate (ESR) were often higher than normal values, while infection did not exist. Claushuis et al. [7] reported that in a study on 189 AS patients, only 52% of patients with higher rheumatic activity presented with high CRP levels. There was no significant correlation between rheumatic activity and CRP level. Therefore, CRP, IL-6, and ESR levels in patients in the present study increased to different extents in preoperative examinations. Based on the exclusion of infections in the oral cavity, respiratory tract, digestive tract, and urinary tract, patients can undergo THA without restoration to normal of the above indicators. No hip infection occurred postoperatively.

In preoperative detection, CRP, IL-6, and ESR levels in patients younger than 40 years old significantly increased. For AS patients in the active stage, comprehensive treatment such as SASP and tumor necrosis factor antagonists should continue after the surgery. It was reported in literatures that in AS patients after THA, the incidence rate of heterotopic ossification was 11–76% [8, 9]. In the present study, patients orally took indomethacin for the prevention of heterotopic ossification. Heterotopic ossification occurred in only three patients (four coxae), and the incidence was 12.9% (4/31); all were Brooker grade II. In addition, joint activities were not affected.

### Choice of surgical approach

An appropriate approach is conducive for exposing the surgical field and helps in the proper placement of the acetabulum. In addition, different operative incisions have great influence on postoperative functions. All hip joints in patients with AS and ankylosed hip joints present with bony fusion. Hence, during THA, the femoral head cannot be dislocated through conventional operation, which may influence the operative field and operative procedure. Therefore, the choice of surgical approach is very important. Through studies on a variety approaches in THA, Sloan et al. [10] concluded that the anterolateral approach could give greater exposure of capsule but it could easily damage the superior gluteal nerve leading to paralysis of tensor fascia lata, as well as affect hip joint abduction function. The posterior approach does not damage the hip joint abductor, and osteotomy can be conducted at the site under the trochanter minor, but postoperative eluxation rate is high. Celiktas et al. [11] considered that posterior approach has an advantage to palpate the sciatic nerve even without dissection, and also, it is easy to release the abductor muscles if necessary. Li et al. [12] considered that anterolateral incision combined with the anterior-posterior approach could well expose the ankylosed hip joint and protect abduction function. This enabled patients to achieve good postoperative recovery. In the present study, the six coxae with severe flexion contractures were treated via the Smith-Peterson incision in the supine position. This procedure was conducive for the full relaxation of anterior tissues in a contracture status. The two coxae with proximal femur deformity were treated via the lateral Hardinge approach, and orthopedic osteotomy of the proximal femoral was performed. The remaining patients (23 coxae) were all treated via the posterior Moore approach, which provided a rapid and thorough exposure of the hip joints, and operation time and intraoperative blood loss were all lesser. Patients in the present study chose an appropriate approach according to specific deformity, which guaranteed the full exposure of the acetabular plane and proximal femoral end, especially the infused boundary between the femoral head and acetabulum. This would be advantageous to the preparation of the osteotomy of the acetabular side and to the relaxation of soft tissues, reducing intraoperative fracture, neurovascular injuries, and other complications.

### Positioning of the acetabulum

The accurate position and angle of the acetabular prosthesis is one of the keys to the success of THA. An inaccurate acetabulum placement may lead to adverse consequences such as high dislocation rate of hip joint, impact, and artificial joint repair [13–15]. During THA operation, the anatomic hallmarks that can be used for positioning of the acetabulum prosthesis include transverse ligament of the acetabulum, location points of pelvic anatomical hallmarks such as the groove between the posterior margin of acetabulum and the sciatic tuber, and the intersection of the lower edge of eminentia iliopectinea, as well as the lateral part of the rami superior ossis pubis, the highest point of the acetabular rim, and the acetabular notch angle [16–20]. However, the accuracy of the manual positioning of the acetabular prosthesis by these anatomical landmarks is low [21]. Intraoperative computer navigation is helpful in the accurate implantation of the acetabulum prosthesis [22]. However, this device is not widely used. We first considered the possible effect of the surgical approach on the acetabular angle in determining the position of the acetabulum prosthesis [23, 24]. For example, if the anterior approach is adopted, the acetabular anteversion can easily become large, which should be corrected during surgery. The boundary where the acetabulum and femoral head infused was first accurately determined, and osteotomy was conducted. The acetabular height was determined based on the abovementioned osseous markers of the pelvis, as well as the upper margin of the obturator foramen and the posterior margin of ischium. Next, the center of the acetabulum was determined according to the femoral head center, which was exposed after femoral neck osteoctomy. The Kirschner wire was driven perpendicular into the surface of the osteotomy, and the center line of the acetabulum was determined by X-ray during the surgery. The anteversion and abduction angles were determined by observing the position of the patient and the angle of the Kirschner wire. Through these methods, the acetabulum can be accurately located. It was found that bony fusion mostly occurred in the weight-bearing area above the hip joint, and soft tissue septa may appear in the bottom part of the acetabulum [25]. Therefore, when reaming the acetabulum, observation is necessary, and the reaming depths were determined by the remaining soft tissues in the bottom of the acetabulum, the acetabular fossa, and the acetabular transverse ligament.

### Selection of hip prosthesis material

Compared with elderly patients, young and middle-aged patients have more activities and a longer life expectancy. Hence, the material for prosthesis is also an important factor. The study conducted by Takenaga et al. [26] suggested that non-bony cement prosthesis was suitable for young and middle-aged patients < 50 years old. In the present study, we found that in some patients with osteoporosis, the hip joint function recovered, and their osseous substance was restored to normal after functional exercise. In the present study, biotype prosthesis was adopted in all patients. During the follow-up, prosthesis fixation was good, and prosthesis loosening and sinking did not appear.

## Conclusions

In summary, after THA treatment, young and middle-aged patients with AS combined with hip bony fusion recovered well. However, in the present study, the number of patients was small, the duration of the study was long, and follow-up duration was short. Therefore, the long-term recovery of joint function and prosthesis survival in some patients needs further observation.

## Data Availability

This data will not be shared, because in recent years, although many scholars have explored this in various aspects, its pathological mechanism remains unclear and there are no standard diagnostic criteria. In order to determine the effective method for preventing and treating this disease, it is necessary to proceed with more large-scale clinical studies.

## Abbreviations

AS: Ankylosing spondylitis
CRP: C-reactive protein
ESR: Erythrocyte sedimentation rate
THA: Total hip arthroplasty
